# A new phenotype of choreic syndrome associating severe freezing of gait and chorea

**DOI:** 10.1002/ccr3.3008

**Published:** 2020-06-02

**Authors:** Brice Laurens, Claire Delleci, Cyril Goizet, Umberto Spampinato, Pierre Burbaud, Etienne Guillaud, Emma Bestaven, Dominique Guehl

**Affiliations:** ^1^ Institut des Maladies Neurodégénératives (IMN) Service de Neurologie CHU Bordeaux Bordeaux France; ^2^ Service de Médecine Physique et Réadaptation CHU de Bordeaux Bordeaux France; ^3^ Service de Génétique Médicale CHU Bordeaux Bordeaux France; ^4^ MRGM INSERM U1211 Université Bordeaux Bordeaux France; ^5^ Neurocentre Magendie Université de Bordeaux‐INSERM U1215 Bordeaux France; ^6^ Service d'explorations Fonctionnelles du Système Nerveux CHU de Bordeaux Bordeaux France; ^7^ INCIA UMR5287 Université Bordeaux Bordeaux France

**Keywords:** chorea, electromyography, freezing of gait, gait

## Abstract

The early onset of gait akinesia should not rule out the diagnosis of hereditary chorea. It would be helpful to proceed to a whole‐genome and long‐read sequencing in order to track a new pathogenic variant including noncoding repeat expansion.

## Graphical abstract

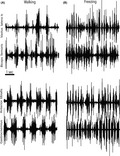

## 1 INTRODUCTION

We report a new phenotype of chorea combining choreic movements and an early freezing of gait (FOG). Genetic screening for the following gene mutations was negative: *HTT* (HD), *JPH3* (HDL2), *TBP* (SCA17), *ATN1* (DRPLA), and C9ORF72. The early FOG episodes could be due to degeneration of the direct striato‐pallidal pathway.

Huntington's disease (HD) is an autosomal dominant disease caused by a mutation of the IT15 gene which is located on chromosome 4 and is responsible for an expanded trinucleotide cytosine‐adenosine‐guanosine repeat sequence.^1^ During the course of chorea, a progressive akinetic‐rigid‐dystonic syndrome appears at the more advanced stages of the disease and masks the choreic movements, which abate dramatically. After obtaining the informed written consent, we report a case of an idiopathic choreic syndrome combining chorea associated with early severe freezing of gait (FOG).

## 2 CASE PRESENTATION

A 66‐year‐old man had experienced choreic movements and falls due to FOG for 6 years. The syndrome was characterized by choreic movements of the face, trunk, and four limbs (see Video [Link], [Link], segment 1) leading to a score of 20/124 on the motor part of the Unified Huntington's Disease Rating Scale (UHDRS).^2^ Gait was dramatically impaired by severe festination and FOG responsible for imbalance and falls 1 year after disease onset (see Video [Link], [Link], segment 2). No segmental akinesia or rigidity was observed in any of the four limbs. Oculomotricity was impaired with interrupted vertical pursuit, as well as swallowing and speech. Cognitive functions were impaired on the mnemonic slope, but verbal fluency and visuo‐constructive functions were preserved. Cerebrospinal fluid was not analyzed, and brain (T1, T2 FLAIR, DWI, T2*) and medullary magnetic resonance imaging showed discrete atrophy of the caudate nucleus, cerebellum, and spinal cord. An autosomal dominant transmission was suspected because his deceased mother had exhibited abnormal movements from the age of 61 years. However, his father, two brothers, three sisters, and three children were symptom‐free. No information on his maternal grandparents was available.

**Figure 1:**
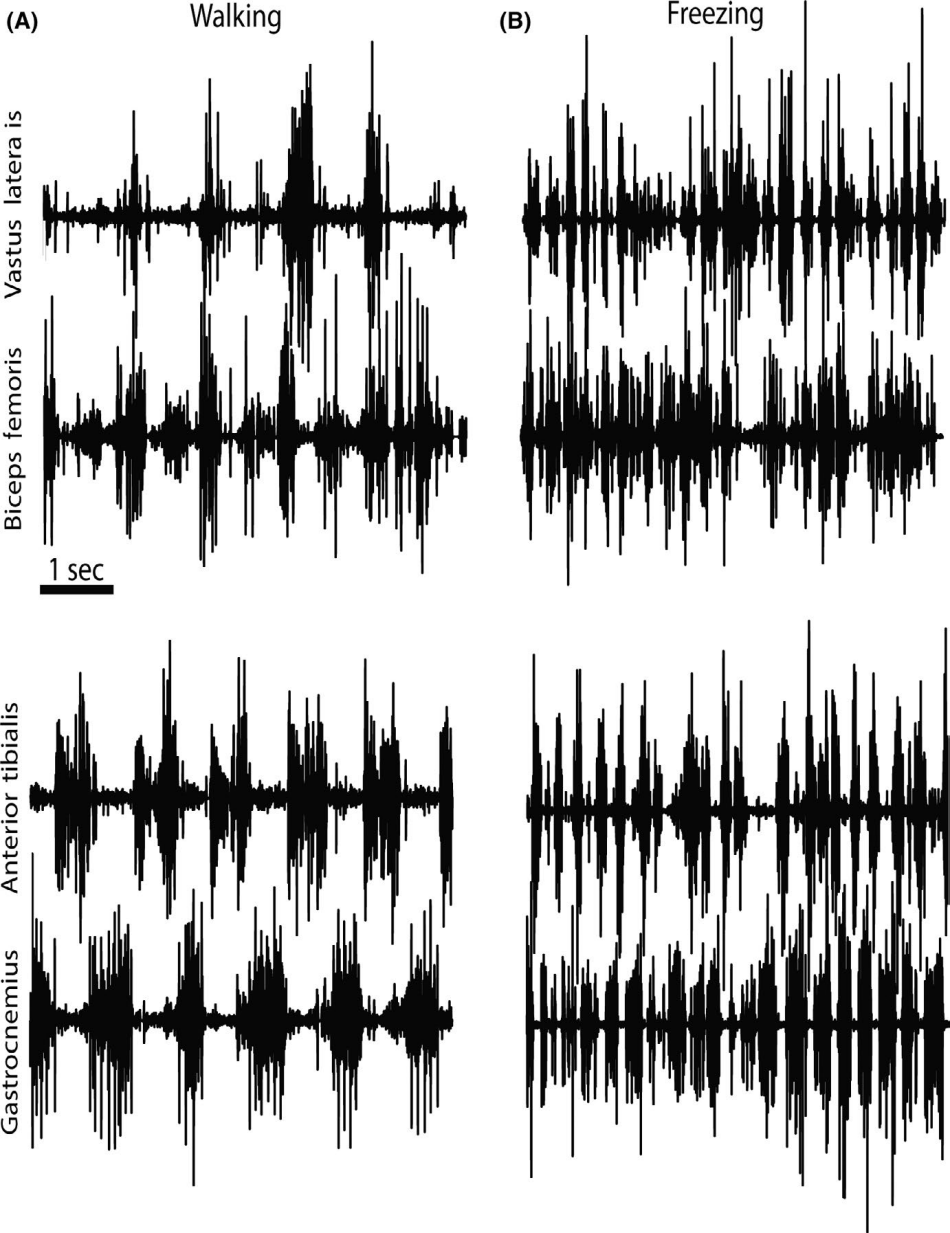
Muscular activation patterns observed during normal gait and freezing of gait. A, Surface electrodes were used to record muscular activity of the left and right (not shown) gastrocnemius, anterior tibialis, biceps femoris, and vastus lateralis during period of gait without freezing. B, Repetitive muscular activity recorded during freezing of gait (FOG). A cocontraction index (CCI) revealed an increase in antagonist muscular cocontractions, especially in the left proximal muscles during the FOG episodes (CCI = 322.0 during gait without FOG vs 2880.1 during FOG; (A,B)). Cocontraction index: *CCI* = (*EMG1 + EMG2*) × *min* [*EMG1 EMG2*]/*max* [*EMG1 EMG2*]

Genetic screening for the following gene mutations was negative: *HTT* (HD) (17‐22 CAG), *JPH3* (HDL2), *TBP* (SCA17), *ATN1* (DRPLA), *VPS13A,* and C9ORF72. In addition, routine pan‐genomic array comparative genomic hybridization (CGH; Human660‐Quad v1.0 DNA Analysis BeadChip; Illumina) revealed no gene dosage abnormalities. A dopamine transporter scan (DAT scan) showed values at the lower limit in the striatum (index for the left and right striatum = 2.19; normal 2.22‐4.17). Electromyographic surface electrodes were used in order to characterize his gait (Figure 1A,B). During the FOG episodes (see Video [Link], [Link], segment 2), a disorganized muscular activity of the left biceps femoris, vastus lateralis, gastrocnemius, and anterior tibialis was observed (Figure 1B).

Levodopa therapy was not tested because no significant dopaminergic degeneration was observed on the DAT scan results and because chorea was disabling, so that we did not take the risk of increasing involuntary movements by introducing dopamine replenish therapy. On the other hand, a treatment with tetrabenazine was initiated at a daily dosage of 12.5 mg and progressively increased up to the dosage of 62.5 mg/d. Despite we observed a significant decrease of chorea with this dosage, FOG and swallowing dramatically impaired and necessitated to stop the therapy.

## 3 DISCUSSION

Although the molecular diagnosis is not yet available, the phenotype of this choreic syndrome is of particular interest because this patient exhibited concomitant dramatic hyperkinetic and hypokinetic symptoms without significant dopaminergic denervation, as attested by the DAT scan results. Such early dramatic akinesia of the lower limbs associated with choreic movements has not been reported previously in choreic syndromes. During the first years of the disease, the medium spiny neurons constitutive of the indirect striato‐pallidal pathway degenerate preferentially, thereby explaining the hyperkinetic syndrome.^1^ On the other hand, the hypokinetic characteristics of gait usually appear as the disease progresses, with degeneration affecting the direct striato‐pallidal pathway and the substantia nigra pars compacta.^3^, ^4^, ^5^, ^6^ At this later stage, the choreic syndrome tends to abate. In our patient, the FOG episodes could reflect a severe akinesia restricted to the lower limbs that cannot be explained by dopaminergic degeneration but is more likely due to early degeneration of the direct striato‐pallidal pathway. Moreover, neither comorbidities nor neuroleptic exposure could explain such gait disturbances.

## 4 CONCLUSION

In summary, this case reveals a new phenotype of chorea combining choreic movements and severe gait akinesia. Its molecular basis remains to be identified as it does not fit any of the gene profiles for the more frequent choreic syndromes. Whole‐genome sequencing and long‐read sequencing to look for noncoding repeat expansion would be highly valuable in familiar neurodegenerative disorders including the clinical situation described here. ^7^

## CONFLICT OF INTEREST

The authors have no conflict of interest to declare.

## AUTHOR CONTRIBUTIONS

DG conceptualized, designed, supervised, and coordinated the study. BL, EG, EB, and DG acquired, analyzed and interpreted the data. BL, CD, CG, US, and DG involved patient treatment and care. BL and DG drafted the manuscript. BL, CD, CG, US, PB, EG, EB, and DG involved in critical revision of the manuscript for intellectual content and approval of the final version for submission.

## Supporting information

Video S1

Sup info

## References

[1] The Huntington's Disease Collaborative Research Group . A novel gene containing a trinucleotide repeat that is expanded and unstable on Huntington's disease chromosomes. Cell. 1993;72(6):971‐983. 8458085 10.1016/0092-8674(93)90585-e

[2] Huntington Study Group . Unified Huntington's disease rating scale: reliability and consistency. Mov Disord. 1996;11(2):136‐142. 8684382 10.1002/mds.870110204

[3] Delval A , Krystkowiak P , Blatt JL , et al. Evolution of locomotion disorders in Huntington's disease. Neurophysiol Clin. 2008;38(2):117‐125. 18423332 10.1016/j.neucli.2008.01.003

[4] Delval A , Krystkowiak P . Locomotion disturbances in Huntington's disease. Rev Neurol. 2010;166(2):213‐220. 19604530 10.1016/j.neurol.2009.05.013

[5] Hwang WJ , Yao WJ . SPECT study of the nigrostriatal dopaminergic system in Huntington's disease. J Neuroimaging. 2013;23(2):192‐196. 22211920 10.1111/j.1552-6569.2011.00671.x

[6] Danoudis M , Iansek R . Gait in Huntington's disease and the stride length‐cadence relationship. BMC Neurol. 2014;14:161. 25265896 10.1186/s12883-014-0161-8 PMC4190343

[7] Ishiura H , Shibata S , Yoshimura J , et al. Noncoding CGG repeat expansions in neuronal intranuclear inclusion disease, oculopharyngodistal myopathy and an overlapping disease. Nat Genet. 2019;51(8):1222‐1232. 31332380 10.1038/s41588-019-0458-z

