# Supplementary material for: A new phenotype of choreic syndrome associating severe freezing of gait and chorea
Source: Clin Case Rep. 2020 Jun 2;8(9):1806–8. doi: 10.1002/ccr3.3008 (PMC7495783; doi:10.1002/ccr3.3008)
Supplement: Supplementary file 2 — Sup info [file CCR3-8-1806-s002.pdf]

Legend to the supporting information video

Segment 1: the patient is sitting on a chair and exhibits choreic movements affecting the face, the trunk and the four limbs; **Segment 2**: the patient tries to initiate gait and exhibits festination and freezing of gait related to left lower limb akinesia; AVI format.
